# Centering Cultural Approaches in Community-Based Participatory Research to Address the Black Maternal Health Crisis

**DOI:** 10.1016/j.focus.2025.100402

**Published:** 2025-08-05

**Authors:** Natalie Hernandez, Dominique Guillaume, Lasha Clarke, Andrea Parker, Sherilyn Francis, Kennedy Lewis, Morgan Davis, Kaprice S. Welsh, Joy Baker, Rasheeta D. Chandler

**Affiliations:** 1 Center for Maternal Health Equity, Department of Community Health & Preventative Medicine, Morehouse School of Medicine, Atlanta, Georgia; 2 Nell Hodgson Woodruff School of Nursing, Emory University, Atlanta, Georgia; 3 School of Nursing, Johns Hopkins University, Baltimore, Maryland; 4 School of Interactive Computing, College of Computing, Georgia Institute of Technology, Atlanta, Georgia; 5 Georgia Obstetrical and Gynecological Society, Duluth, Georgia; 6 WellStar Health System, Atlanta, Georgia

**Keywords:** Maternal health, women’s health, maternal morbidity and mortality, community-based participatory research, health equity

## Abstract

**Introduction:**

Black women in Georgia are more likely to die from pregnancy-related complications during the postpartum period than other their non-Black counterparts. Developing effective solutions to address this public health crisis requires strengthened partnerships between researchers and community-based organizations, with the voices of Black women being at the center of the research and innovation. This study examined perspectives of community-advisory board members on a community-based participatory research approach that addresses adverse postpartum health outcomes for Black women living in rural Georgia.

**Methods:**

This qualitative descriptive study employed semistructured qualitative interviews with members on the community-advisory board and research team (N=16) consisting of all Black women. Thematic analysis was used to analyze data deductively, with codes being categorized into overarching themes.

**Results:**

Themes that were elicited from interviews included community; culture; acceptability of the Positive Outcomes, Research Recommendations, Community Collaborations, and Honorable delivery of healthcare research approach; and Black maternal health disparities. Participants voiced the importance of the inclusion of Black cultural aspects within maternal health interventions and the importance of providing Black women with a platform to share their perspectives and experiences. Participants provided insight on promoting Black maternal health equity and reducing power dynamics within research.

**Conclusions:**

Centering culture and community and using Black women’s experiences to guide the development of research interventions will be critical in developing relevant solutions to address the Black maternal health crisis. Research should be designed with the input of target communities while supporting synergistic relationships between communities and researchers.

## HIGHLIGHTS

• Community-based participatory research (CBPR) can address Black maternal health disparities.
• CBPR that is culturally informed mitigates the subject–object binomial in CBPR.
• Black maternal health researchers must ensure that Black women are represented in the research process.

## INTRODUCTION

The current maternal morbidity and mortality rates for Black women in the U.S. are staggering, with Black maternal mortality being considered to be a public health crisis.^1^ Black women in the U.S. are between 3 and 4 times more likely to die from pregnancy-related complications than their White counterparts.^2^^,^^3^ The state of Georgia in particular has one of the highest maternal mortality rates in the nation, with the death rate among Black women in Georgia at 66.3 deaths per 100,000 live births.^4^ This rate is significantly higher than the national maternal death rate for all women (29.6 deaths per 100,000 live births).^4^ Black women living in rural areas of Georgia are more likely to die from pregnancy-related causes throughout their pregnancy and up to 1 year after delivery.^5^^,^^6^ Black women in rural Georgia are disproportionately susceptible to maternal morbidity and mortality, particularly because they are the most difficult to reach.^7^, ^8^, ^9^, ^10^ Given this public health crisis, health departments and healthcare providers throughout Georgia have placed a heightened focus on implementing interventions that address health disparities during the postpartum period (e.g., mobile clinics, home visitation programs).^5^ Although these programs may prove to be effective in identifying and addressing certain health conditions and needs during the postpartum period, their geographic reach has been severely limited, particularly within rural communities in Georgia where Black women experience the highest rates of adverse maternal health outcomes.^2^^,^^8^^,^^9^^,^^11^^,^^12^ Furthermore, many programs that have been developed to address disparities in Black maternal health outcomes during the postpartum period have been developed without the input of key members of the community. This is concerning because the lived experiences, unique expertise, skills, and perspectives of Black mothers should be at the center of all research questions and interventions to successfully address Black maternal health disparities.^13^

Addressing maternal health inequities among Black women requires re-evaluating traditional methods of research, which largely has not included their voices in developing solutions to this public health crisis.^13^^,^^14^ Community-based participatory research (CBPR)—a collaborative approach that fosters collaboration between scientific researchers and community members—offers considerable potential in addressing Black maternal health disparities and dismantling oppressive structures that contribute to adverse health outcomes.^15^, ^16^, ^17^ CBPR highlights team science by capitalizing on the strengths, perspectives, and expertise of individuals from diverse backgrounds who serve as advocates for their community with the inclusion of community advisory boards (CABs), which often serve as a source of leadership and structure to guide research and implementation activities.^17^ It has been readily acknowledged that CABs are essential in advocating for the communities in which they are based in within research studies. However, despite the importance of CABs in research, most of what is known about CABs are described in articles about the research project itself.^18^ As of date, very little is known on the perspectives of CAB members who participate in research, particularly within the field of Black maternal health.

Although CBPR has gained momentum over the last decade in health disparities research, few studies have been led by Black researchers and have adequately showcased the perspectives of CAB members in the use of CBPR to address Black maternal health inequities. The purpose of this study is to present CAB members’ perspectives of the CBPR approach, which is being used in the authors’ parent study—Preventing Maternal Mortality using Mobile Technology—that focuses on mobile health technology to provide access to postpartum care for Black women in rural Georgia.^19^ The authors have termed their CBPR approach Positive Outcomes, Research Recommendations, Community Collaborations, and Honorable delivery of healthcare (PORCH). The team elicited perspectives from CAB members along with members of the research team regarding how the approach can be used to address disparities in Black women’s postpartum health. Results from this study, along with those from the parent study, can guide future research interventions that employ CBPR approaches to address health disparities within Black communities.

## METHODS

The PORCH approach embodies principles of community engagement and cultural responsiveness. It emphasizes fostering trust, prioritizing cultural understanding, and addressing systemic inequities through collaborative, community-informed strategies. Studies have associated Black women’s race, language, and culture with a nearly 3 times higher odds of discrimination.^20^ Improving quality in obstetrics care for Black women requires centering the determinants of race and culture because misconceived prenotions of race and culture on behalf of health systems and providers can lead to hinderances in obtaining quality maternity care. Rooted in phenomenology and critical theory, PORCH recognizes the historical and structural barriers that impact Black maternal health. Critical theory aims to provide an intersubjective and in-depth perceptions of the social world and challenges the societal structures that perpetuate health and social disparities among individuals and communities.^21^ Critical theory recognizes diverse contributions and analyzes different forms of inquiry within specific methodologies and research problems and is essential to understanding the distributions of health and disease in diverse populations.^13^^,^^21^ Phenomenology provides researchers with an understanding of the lived experiences of the patients and communities that they work with. More specifically, Husserl’s phenomenology emphasizes that natural science alone does not provide a complete understanding of the human experience.^22^^,^^23^ Integrating critical social theory with phenomenology can be beneficial for researchers who are interested in examining health disparities and the historical, societal, political, and economic factors that perpetuate adverse health outcomes among Black women.^24^ Unlike traditional CBPR models, PORCH situates Black cultural values and lived experiences at the core of its framework. Through this culturally nuanced approach, it aims to dismantle power dynamics, center the voices of Black mothers, and catalyze systemic change.

### Study Sample

CAB members were purposefully selected through an environmental scan involving gathering and interpreting information from internal and external organizational environments. This process entailed analyzing cultural, socioeconomic, linguistic, technological, and geographic environments.^25^ The research team was vigilant regarding selecting organizations that were motivated to foster social change and sustainability. The team also selected organizations that were well trusted in the communities that they served given the historical distrust of medicine and research among Black communities. Through the environmental scan, the research team determined current needs and assets with local leaders and organizations in rural Georgia and identified potential CAB members who were well versed in the needs of the priority community. CAB members consist primarily of rural Black postpartum women, researchers, healthcare providers, community-based health advocates and leaders, and policy experts across a range of disciplines who are representative of the priority community.

### Data Collection and Analysis

All CAB members and researchers participated in qualitative interviews. Because this was a qualitative descriptive study, in-depth interviews were used for data collection to discover the who, what, and where of phenomena, per Sandelowski (2000) guidelines.^26^ Interviews lasted approximately 45 minutes and were conducted over Zoom.^27^ Questions on the semistructured interview guide asked about community, culture, representation, and significance of the PORCH approach, how the approach could address Black maternal health disparities, and how the approach could be effective in a virtual environment (Appendix 1, available online). Items on the interview guide were informed by the philosophical underpinnings of the study with an emphasis placed on culture and community in the context of maternal health.^28^ Interviews were conducted by 2 members of the research team (KL and MD) and recorded and transcribed using Otter.ai software.^29^ Transcripts were checked for accuracy against the recording and uploaded on Atlas.ti software for data analysis.^30^ Data were analyzed using a qualitative descriptive approach for data analysis. Two members of the research team (DG and KL) conducted open coding on 3 randomly selected transcripts using deductive codes generated from the interview guide in addition to inductive codes (Table 1). Afterward, codes were discussed, and a codebook was developed following MacQueen et al.^31^ guidelines. The codebook was used to code the remaining transcripts, and edits to the codebook were made iteratively. Discrepancies in coding were discussed among the team members until consensus was achieved. Results from the analysis were shared with participants for informant feedback and member validation to ensure that the perspectives of participants were adequately captured and portrayed. This study was approved by Morehouse School of Medicine IRB (Protocol Number 177446).

**Table 1:** Coding Framework

Theme	Open code
Community	
	Positive aspects of community
	Negative aspects of community
Culture	
	Linking culture to the PORCH
	Positive aspects of culture
PORCH approach acceptability	
	PORCH approach benefits
	PORCH approach significance
	PORCH approach experiences
	Equalizing effect
Black maternal health	
	Addressing health disparities
	Improvement of health outcomes
	Fostering sustainability
	Translating research into impact

PORCH, Positive Outcomes, Research Recommendations, Community Collaborations, and Honorable delivery of healthcare.

## RESULTS

A total of N=16 participants completed interviews (*n*=8 CAB members and *n*=4 researchers) (Table 2). The authors identified 4 emerging themes that encapsulated Black women’s perspectives about the PORCH approach and how it can be used as an approach to address Black maternal health disparities by informing the development of culturally tailored interventions in the Black community.

**Table 2:** Participant Characteristics

Race/ethnicity	Geographic area of origin	Role	Expertise
Black/African American	Georgia (rural)	CAB	Black maternal health, community advocacy
Black/African American	Florida (rural)	Researcher	Black maternal health, HIV prevention
Black/African American	Georgia (rural)	Researcher	Black maternal health, community advocacy
Black/Caribbean American	Haiti (urban)	CAB	Sexual and reproductive health, HIV prevention and treatment
Black/African American	Georgia (rural)	CAB	Black maternal health, obstetrician/gynecologist
Black/African American	California (urban)	Researcher	Digital health, human-centered design, health disparities
Black/African American	New York (urban)	CAB	Black maternal health, community advocacy
Black/African American	Georgia (rural)	CAB	Family and Black maternal health
Black/Caribbean American	New York (urban)	CAB	Black maternal health
Black/African American	Georgia (rural)	CAB	Black maternal health
Black/African American	Louisiana (rural)	CAB	Black maternal health, community advocacy
Black/African American	Virginia (rural)	Researcher	Black maternal health, Health disparities, health equity

CAB, community advisory board.

Participants vividly described the communities in which they were raised and compared their experiences with those of the communities in which they currently live. The majority of participants described having strong social networks and enjoying the communities in which they were raised in. In terms of favorable aspects of their current community, several women highlighted the tight-knit feeling of their community as one of the determining factors of their decision to reside there, the heightened level of interaction they experience with their neighbors as a favorable community aspect, and the diversity: “So just the close proximity of everyone and having your friends right there, my best friend was next door, my other friends was upstairs. So just having, like, everybody just right there all the time, other families, the closeness of everything your family knew someone else's family.” Said a CAB member. A researcher said, “I love Atlanta, I love the diversity of Black people…I think it shows how Black people are not a monolith and there's just so much diversity of Black thought, Black experience, Black love, that I really enjoy.”

However, in terms of unfavorable aspects, several CAB members struggled to engage with their communities and described interactions as laborious and inorganic. A lack of opportunities for community residents was also listed as an unfavorable aspect. In addition, certain CAB members emphasized racism and poverty within their communities as unfavorable aspects that they wished to change.

The racism and the poverty… they're very intersectional. Because the racism that exists here in southwest Georgia is so thick and so rampant. And it's the reason why there is so much poverty, because of the systems that have been created by people that want to maintain racist structures. So, it's hard for me to separate those two, like all of our work is rooted in the intersection of racism and poverty. So, if I could eradicate that, I think [city in rural Georgia] would be an amazing place to live. –A CAB member

I think that I would actually change the level of opportunities that are available for folks [in my community]. –A CAB member

In developing the PORCH approach, which is reflective of Black women in the community, the importance of understanding thoughts and perspectives toward Black culture was recognized. Interviewees discussed favorable and unfavorable aspects of the culture in which they identified and were asked about the relevance of culture in the context of Black maternal health. Nearly all participants emphasized the importance of community and maintaining strong social networks, particularly in addressing Black maternal health inequities.

Participants viewed the PORCH as functioning as a platform to promote community and social networks that were in line with Black culture.

When I heard it [about PORCH] initially, the first thought that came to my mind was the porch was where as a little girl, I was protected. And as I got older, I protected my younger cousins, and taught them things. And now my generation is starting to take over. When I thought about that, in terms of Black maternal health, I thought we have to stand in the gap for these moms and we need to be there to support and to educate and to have a space so that they can feel safe… so they can be able to talk and have somebody listen and know that that there is a place of safety… –A CAB member

The importance of culture and connecting to one’s roots was significant in addressing Black maternal health disparities as described by the following participant:

I like the Afro-centric worldview [of the PORCH approach]…a lot of that is going back to our roots. I think the PORCH offers an opportunity to really recognize how much the roots of our culture, our, upbringing, our strengths that we have, as Black people, needs to be a part of the conversation to approach health and equity…that really is a part of healing. –A CAB member

Participants elaborated on what PORCH represents for them, with women describing it as a safe space that promoted authenticity and support for Black women to discuss health issues that mattered to them. One participant reiterated the need to place Black mothers in a nonthreatening environment that takes their specific needs and cultural values into consideration when providing them with healthcare services. Several participants stated that the approach should be extended into more Black communities to improve maternal health outcomes:

I mean, I think the porch represents a culturally safe space to be able to be authentic. Right and true. I think that I love the idea of the porch. Because it's, I think, yes, it becomes that space, that safe space to be free and authentic, to discuss and talk about, what brings you joy, what brings you hurt, what brings you you know, anger, what I mean, I think it's just a place to be authentic and genuine, in your conversations in your dialogue. Without being judged. –A CAB member

We have to stand in the gap for these moms and we need to be there to support and to educate and to love and to, you know, be able to, to have a space for them so that they can feel safe…...there's somebody that can give them advice that is credible, that, there is that place. –A CAB member

An emphasis was placed on the equalizing effect of the PORCH approach, with CAB members voicing that it eliminated the physician–patient or subject–object power dynamics as described by several participants.

So I feel like having this approach and then having it led by Black women will bring a sense of comfort to individuals, and they'll be willing more willing to actually listen to the information…these researchers, they take the time to explain it to you so that you can understand explain to you on your level. And that's one thing that I really appreciate about them. So I feel that you know, just being on their level talking to them, explain everything to them by telling them preventive measures and coming from another Black woman. I think that'll be very, very, very beneficial. –A CAB member

Bringing all those people onto the porch is really, I think, what we need to do, and in a way that surrounds the mom in an atmosphere that's non-threatening, where she feels like she has access to these people, and they're not in some high rise building that she can never reach, or get to people that she can't talk to that seem to be so much higher, or mightier, like in this power perception that patients sometimes tend to have of us. –A CAB member

Participants discussed how they anticipated that this approach would impact Black women’s health. The majority of responses were optimistic, were hopeful, and emphasized the need for more research approaches to be tailored toward the needs of Black women. There was a consensus that Black women’s health would improve if research efforts targeting their communities incorporated Black culture, Black imagery, and Black values into marketing and recruitment plans. The PORCH research approach, they argued, was a great attempt at fostering an inclusive, Black culture–specific approach developed for Black women by Black women.

I love that it's Black women and women of color, taking care of Black women and addressing Black issues that are serious issues… Black maternal health is, is a major issue right now. And it should not be something that we're dealing with in America, but we are. And so to have, I think a group of women that are so brilliant creating these studies, and doing this research to help other Black women, it's amazing to be a part of, and I think the name is so fitting. –A CAB member

The lack of health-related knowledge that Black women have regarding their reproductive and postpartum health was mentioned as a key contributor to their adverse health comes. CAB members stated that maternal health education should be delivered to Black women in terms that they can relate to and easily understand as indicated by the following respondent:

Because a lot of women don't know a lot about … health disparities and how to, like overcome health disparities, preventable measures…and that's one thing that I really appreciate about [these researchers]…[women] being on their level when talking to them, [the researchers] explain everything to them [women] by telling them preventive measures …and this is coming from another Black woman. I think that'll be very, very, very beneficial. –A CAB member

Solutions to addressing Black women’s health disparities through the PORCH approach included using the structural PORCH as a symbol of unity and placing Black women at the forefront of these research efforts. Several participants felt that previous research efforts aimed to address Black women’s maternal health outcomes were White male led, which contributed to a delay in the translation of the research findings into tangible improvements. CAB members emphasized that interventions to address health disparities for Black women should be developed and led primarily by Black women.

No one knows Black women better than Black women. And so I think no one is better positioned to create initiatives and create solutions better than other Black women and women of color. So I think the approach of again, as taking care of our own community, is key. –A CAB member

You know, this is one of my first times being a part of something where it's all Black leaders. I've always been in a number of spaces where I'm the only one [Black researcher] and even just the creation of the symbolism of PORCH is big for me, and a sense of like, "Hey, I actually am doing something that sits in my truth," and not necessarily trying to fit into some Eurocentric standard of wellness. –A researcher

The diverse professional backgrounds of CAB members and researchers was mentioned as a benefit of the PORCH approach given that successfully addressing Black maternal health will require a multidisciplinary team effort. This was detailed by a CAB member who is an obstetrician/gynecologist and voiced the need for multidisciplinary efforts in reducing health disparities for Black women.

Yes, I'm an OB, and I am, you know, certainly qualified to take care of medical care during pregnancy. But I'm not a psychiatrist, I'm not a social worker, I'm not a pediatrician. I'm not the "Be-all-end-all." So there's so many more things that have to come, you know, around this mom in order for her to have like a complete level of support. And I think that for so long, we've, we've missed that we've missed the fact that this is a team sport… we need to make sure that she can have access to care before she gets pregnant…Because if she comes to me with so many problems, I'm already behind the eight ball... –A CAB member

## DISCUSSION

This study captured CAB members’ perspectives of CBPR approach, PORCH, and its potential impact on addressing Black maternal health in rural Georgia. Participants strongly endorsed the PORCH approach largely owing to the cultural relevance and representation it provided. An important finding was how the approach functioned as an equalizer, helping to overcome subject–object power dynamics commonly present in CBPR research.^32^^,^^33^

The need for a heightened focus on culture and social context has been cited in addressing maternal health disparities. However, few studies and interventions have examined the influence of cultural and social depictions that perpetuate racial inequities.^15^ To date, a deep commitment to cultural humility and understanding—expressed through the development of mutually respectful, dynamic partnerships with communities—is often lacking.^34^^,^^35^ Our study underscored the importance of Black researchers leading studies related to Black maternal health. Women in the study strongly advocated for Black women taking the lead in addressing Black maternal health issues, which contributed to their endorsement of the PORCH approach.

There is limited research evaluating the impact of Black researchers leading studies on Black communities. Sealy-Jefferson^15^ emphasized the need for research focused on oppressed communities to be led by members of those communities as equal thought partners. She also called for the liberation of research from subject–object power dynamics and the promotion of participatory research that can inform action-oriented methodologies.^15^ Integrating traditional Black images, messages, cultural values, and perspectives into interventions targeting Black women can allow them to identify with the intervention’s objectives and feel more inclined to participate in research.

To address maternal health inequities among Black women, it is imperative to re-evaluate and challenge traditional research methodologies that have largely excluded Black women’s voices in developing solutions to this public health crisis.^13^^,^^14^ Current Black maternal health research, often led predominantly by White researchers, may reflect biased perspectives and cultural worldviews. This disconnect can result in an inadequate understanding of the social, cultural, and clinical determinants of health for Black women.^13^ The PORCH approach, developed by Black researchers and involving a team of Black women from diverse backgrounds, offers a culturally grounded framework for creating community-based solutions.

This approach explicitly acknowledges the structural and systemic factors contributing to the misappraisal of Black women’s clinical risk factors. It emphasizes the need to consider these broader contexts when seeking to improve health outcomes. Future research should adopt approaches similar to PORCH, ensuring Black women’s representation throughout the research process to establish synergistic partnerships that foster improved community outcomes. Health research, information, and public health interventions should be delivered in a manner that aligns with Black cultural values.

### Limitations

Owing to the limited sample size, the findings from this study are not generalizable. Responses to the PORCH interview questions may not reflect the views of all members of the Black community. However, the goal was not generalizability rather the collection of rich, in-depth data on an understudied phenomenon. Social desirability bias may have influenced participant responses. In addition, the authors did not conduct member checking, which could have enhanced the validity of the findings. Despite these limitations, this study is among the few to employ a culturally centered, theory-informed CBPR approach to address Black maternal health disparities.

## CONCLUSIONS

Centering culture and community and elevating Black women’s leadership in addressing maternal health issues are critical to developing effective, relevant solutions for the Black maternal health crisis. This study highlights the importance of designing research with input from target communities and fostering strong, reciprocal relationships between researchers and community members. The findings can inform future CBPR interventions aimed at addressing maternal health disparities in Black communities. Future studies should ensure that CAB members play a leading role in the research process and that their perspectives are continuously integrated to align interventions with community needs and values.

## Appendix Supplementary materials

Image, image 1

## Footnotes

Supplementary material associated with this article can be found in the online version at doi:10.1016/j.focus.2025.100402.
