# Supplementary figures and images for: Centering Cultural Approaches in Community-Based Participatory Research to Address the Black Maternal Health Crisis
Source: AJPM Focus. 2025 Aug 5;4(6):100402. doi: 10.1016/j.focus.2025.100402 (PMC12509760; doi:10.1016/j.focus.2025.100402)

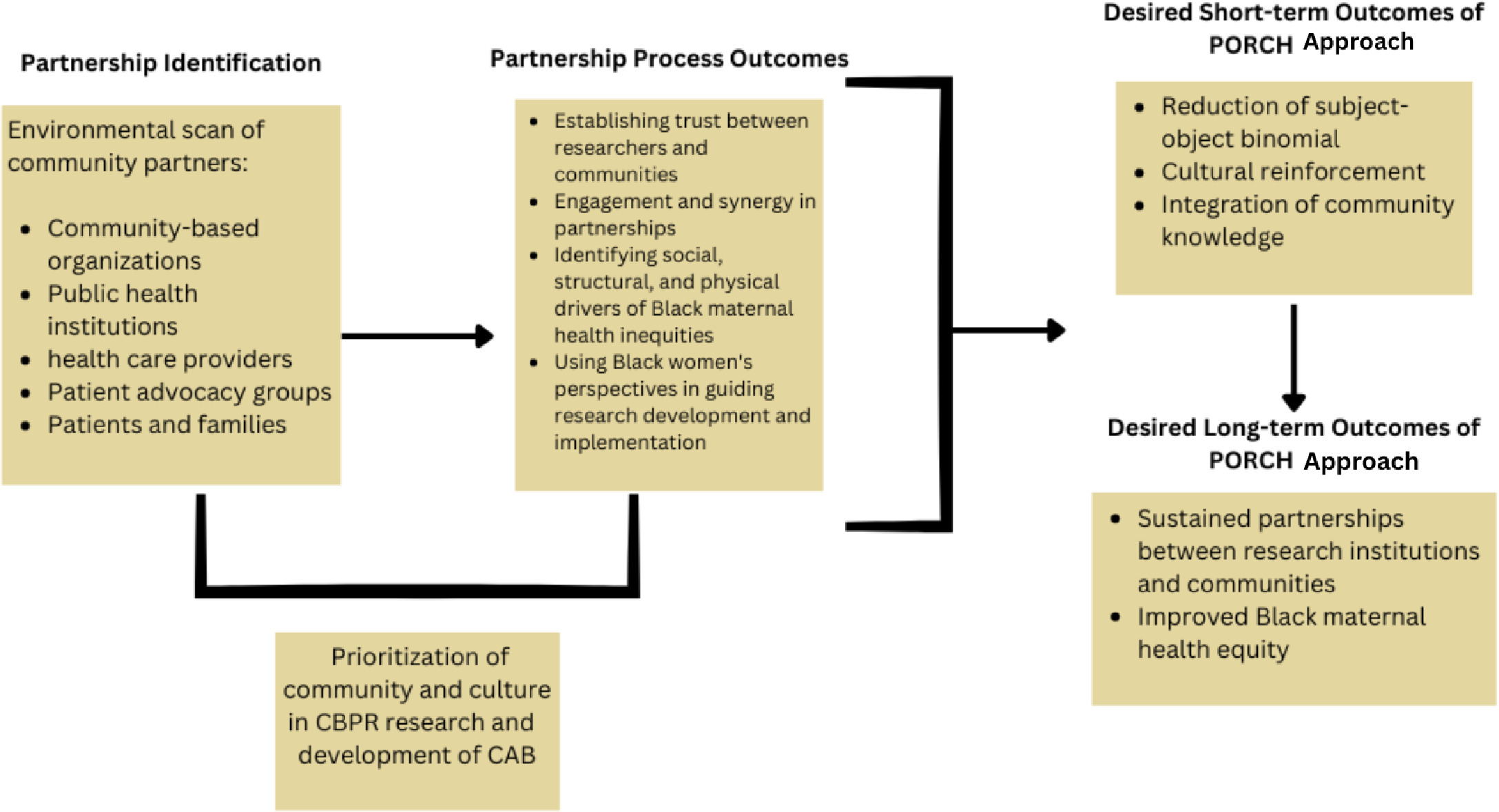

Supplement: Supplementary file 1 [file mmc1.jpg]
